# When Overdose Strikes: Successful Use of Hemodialysis in Valproic Acid Toxicity

**DOI:** 10.7759/cureus.87534

**Published:** 2025-07-08

**Authors:** Stella Zaringhalam, Harpreet Dosanjh, Waleed A Awan, Arian Gower, Jasprit Takher

**Affiliations:** 1 Internal Medicine, Los Robles Regional Medical Center, Thousand Oaks, USA

**Keywords:** general internal medicine, intensive care medicine, medical toxicology, nephro critical care, pulmonary critical care

## Abstract

Valproic acid (VPA) toxicity is a serious and potentially life-threatening condition that can arise from overdose or impaired drug metabolism. While mild cases may present with central nervous system depression, severe toxicity can lead to coma, multi-organ failure, and death. This case report discusses a 21-year-old male with a history of schizoaffective disorder, polysubstance abuse, and suicidal ideations who presented with altered mental status after a suspected overdose of VPA. His serum valproate level was found to be 879 µg/mL, significantly exceeding the therapeutic range. Despite initial supportive care, including intravenous fluids, activated charcoal, and L-carnitine, the patient’s condition failed to improve. After consultation with toxicology, neurology, and nephrology teams, the decision was made to initiate continuous renal replacement therapy (CRRT) to expedite the removal of valproate. The patient’s mental status gradually improved, and he was successfully extubated after three days of hospitalization. This case highlights the importance of early recognition, aggressive management, and the potential role of hemodialysis in improving outcomes in patients with severe VPA toxicity. Timely intervention can significantly reduce mortality and morbidity associated with VPA overdose.

## Introduction

Valproic acid (VPA) is a widely used anticonvulsant and mood stabilizer, prescribed for the management of epilepsy, migraine attack prevention, bipolar disorder, and other various psychiatric conditions [1]. Its mechanism of action is multifaceted. While generally safe when used appropriately, one of its key effects is the inhibition of voltage-gated sodium channels, which reduces neuronal excitability and prevents the rapid firing of action potentials. Additionally, VPA increases the levels of gamma-aminobutyric acid (GABA), an inhibitory neurotransmitter, by enhancing its synthesis and inhibiting its breakdown. This GABAergic effect contributes to the drug's calming effect on the central nervous system [2]. VPA also affects histone deacetylases, leading to changes in gene expression that may help stabilize mood and exert neuroprotective effects. 

VPA toxicity can manifest in a range of symptoms, affecting multiple organ systems, and is a serious concern, especially in cases of overdose or prolonged use. VPA toxicity can present with various neurological and systemic manifestations, including confusion, lethargy, altered mental status, and, in severe cases, coma and multi-organ failure [2]. Hepatotoxicity and neurotoxicity are the most significant known risks, which can present as jaundice, fatigue, abnormal liver function tests, drowsiness, cognitive impairment, and tremors [2]. Mild toxicity is classified as a serum VPA level of 100-150 µg/mL (Note: these levels can vary slightly depending on the laboratory and the clinical context). Moderate toxicity accounts for VPA levels of 150-200 µg/mL, and severe toxicity is classified as VPA levels >200 µg/mL [3]. Management of mild-moderate VPA toxicity includes immediate supportive care with hydration and antiemetics, and close monitoring of the patient's clinical status and laboratory values would be sufficient. For severe toxicity, immediate medical intervention is required, often in an intensive care unit (ICU) setting [4].

However, in cases of severe toxicity, especially when accompanied by persistent altered mental status or organ dysfunction, more aggressive interventions may be required. Although rare, hemodialysis (HD) or continuous renal replacement therapy (CRRT) has been shown to be effective in expediting the removal of valproate from the system, thereby improving patient outcomes in cases of life-threatening toxicity [3,5]. This case report highlights a patient with valproic acid toxicity who required intubation and dialysis, which resulted in mental status improvement. 

## Case presentation

A 21-year-old male with a history of schizoaffective disorder, suicidal ideations, and polysubstance abuse presented to the emergency department with altered mental status. The patient was being brought into the emergency department by staff of an alcohol and depression rehabilitation facility, where they found empty bottles of levetiracetam, valproate, and benzodiazepines near the patient. The facility was regularly doing drug screening tests, and all medications were accounted for, so other intoxicant workups were deferred due to staff being present in the emergency department with the patient. Vitals were significant for a temperature of 97.5 degrees Fahrenheit, a heart rate of 110 beats/minute, blood pressure of 123/89 mmHg, and oxygen saturation of 88% on room air. Physical examination revealed a lethargic patient with a Glasgow Coma Scale (GCS) score of 7 (E2, V2, M3). Lungs were clear to auscultation bilaterally, the abdominal exam was benign, and heart sounds were present without any murmurs. The electrocardiogram showed sinus tachycardia without any significant T wave abnormalities. Initial ABG showed pH 7.2, CO₂ 67, and HCO₃ 24.

Given the severity of his symptoms and the patient's mental status, the patient was promptly intubated for airway protection. Pupils were assessed post-intubation by the emergency department, which noted delayed pupillary response and mydriasis bilaterally. Initial laboratory results revealed a significantly elevated serum valproate level of 879 mcg/mL (normal therapeutic range 50-100 mcg/mL), along with liver enzyme elevation (AST 140 IU/L, ALT 118 IU/L) and normal renal function (Table 1). A head CT scan was unremarkable, and no signs of acute stroke or mass effect were seen. Given the high valproate level and the patient’s altered mental status, valproic acid toxicity was suspected. The patient was initially treated with aggressive intravenous fluids, glucose, thiamine, naloxone, and activated charcoal (given the recent ingestion) and supportive care. 

**Table 1:** Patient admission laboratory values

Laboratory Tests	Patient Laboratory Value	Reference Laboratory Values
Hemoglobin, g/dL	14.1	11.7-15.7
White Blood Cell	7.4	4.0-11.0
Platelet	273	150-400
Sodium, mmol/L	139	136-145
Potassium, mmol/L	3.9	3.6-5.1
Bicarbonate, mmol/L	15	21-32
Creatinine, mg/dL	1.37	0.55-1.02
Alkaline Aminotransferase, units/L	140	10-37
Alanine Aminotransferase, units/L	118	7-55
Glucose, mmol/L	108	80-140
Valproic Acid, mcg/ml	879	50-100
Lactic Acid, mmol/L	7.0	0.4-2.0

The CDC was contacted for further recommendations. Based on their guidelines, the patient was initiated on L-carnitine intravenously every 6 hours and activated charcoal 25 g every 4 hours. However, the patient’s mental status and blood valproate levels failed to improve based on this regimen (880 mcg/mL). After consulting with the toxicology, neurology, and nephrology teams, the decision was made to initiate hemodialysis (about 10 hours after admission) to expedite the removal of valproate from the patient's system. Valproate is known to be dialyzable, and dialysis has been shown to improve outcomes in cases of severe toxicity [3]. The patient underwent continuous renal replacement therapy (CRRT), which resulted in a gradual improvement in his mental status. Valproic acid levels subsequently were checked and decreased to 518 and 310 within 12 hours after hemodialysis. Over the course of the next 24 hours, the patient’s GCS improved from 7 to 15, and he began to respond to verbal stimuli and follow commands, and was successfully extubated on day three of admission. The patient was transferred to the medical/surgical unit and ultimately discharged to a psychiatric hospital for an apparent suicide attempt.

## Discussion

Valproate toxicity is a potentially fatal condition that can arise from an overdose or impaired drug metabolism. Valproate, also known as valproic acid, is a branched-chain carboxylic acid introduced as an anticonvulsant in 1978, primarily used to treat partial and generalized seizures [1]. While acute valproate intoxication often leads to mild, self-limiting central nervous system depression, severe toxicity and even death can occur in some cases.

VPA overdose typically presents with gastrointestinal symptoms such as nausea and vomiting, followed by central nervous system manifestations including headache, ataxia, altered levels of consciousness, confusion, and, in severe cases, coma [4]. When serum levels exceed 850 μg/ml, more severe complications such as hypotension, cardiac arrest, and respiratory depression requiring intubation have been documented [3]. The coma observed in VPA overdose is multifactorial, with contributing factors including hyperammonemia, high anion gap lactic acidosis, direct sedative effects, cerebral edema, and mitochondrial dysfunction [4]. Additionally, both overdose and therapeutic dosing of VPA can lead to complications such as pancreatitis, elevated hepatic transaminases, and bone marrow suppression. Laboratory findings often include hypernatremia (due to the sodium salt of valproate), hypocalcemia, dysphosphatemia, and an elevated anion and osmolar gap [2,3,5]. 

The management of sodium valproate overdose primarily involves supportive care. Administration of oral activated charcoal at a dose of 1 mg/kg may be beneficial in reducing gastrointestinal absorption, particularly if administered within the first hour of ingestion [1,6].

Hemodialysis, hemoperfusion, and their combination have all been shown to effectively reduce serum valproate levels. In the outpatient setting, we used hemodialysis to achieve this goal. Due to its relatively low molecular weight (144.2), valproate is highly dialyzable, though this is somewhat counteracted by its high protein binding [3,7]. A meta-analysis revealed that low-efficiency or low-flux hemodialysis (HD) removes only approximately 20% of the administered dose at therapeutic levels. However, at toxic concentrations, where plasma protein binding sites are saturated, dialysis can significantly remove valproate [5]. Both high-flux HD and hemodiafiltration (HDF) have proven effective in reducing serum valproate levels in both toxic and therapeutic conditions [8].

Indications for renal replacement therapy in cases of sodium valproate intoxication include plasma levels exceeding 700 μg/ml, rapid clinical deterioration, signs of hepatic dysfunction, and ongoing drug absorption [3,5].

In conclusion, the use of renal replacement therapies seems the most effective treatment for valproate overdose and should be strongly considered, besides other supportive therapies in a patient with metabolic encephalopathy, refractory coma, and serum valproate levels above 700 μg/dl [3,5,9].

## Conclusions

This case illustrates the importance of early recognition and aggressive management of valproate toxicity. While most cases of valproate toxicity can be managed with supportive care, severe cases may require intubation and dialysis for optimal recovery. Clinicians should be aware of the potential for serious toxicity, especially in patients with noncompliance, drug interactions, or renal impairment. Timely intervention, including the use of hemodialysis, can significantly improve patient outcomes in cases of severe valproic toxicity. 
